# An Interesting Correspondence

**Published:** 1856-02

**Authors:** 


*An Interesting Correspondence.*—Every one has noticed that he sometimes becomes deeply interested in a person, or a name even, without any assignable cause. Some mysterious sympathy, some unknown exercise of the "odic force," compels a feeling of interest which we do not possess for those nearer to us in the relations of life. Such was our case, when, on assuming the ownership of the Journal, we found, (among others) the names of two individuals were marked as in debt to the concern since No. 1, vol. 1.

In a moment our fancy took wings. We thought of the one hundred and twenty successive months during which the Journal had been regularly mailed to these men, the mails had regularly conveyed them, and the poor books themselves had gone to these undiscovered countries from which no

Journal e'er returned. Who and what are these subscribers? we asked ourselves. Are they veritable men and doctors? walk they the earth, give they physic, possess they pill-bags? Or are they myths, unrealities, metaphysical illusions, deceptions of the Evil One, mere *nominis umbra?*

We determined to find out. We wrote them letters, and repeated that operation regularly every Saturday night for three consecutive months. No answer came, but in the course of the summer we found ourselves within twelve miles of the address of one of them. We appealed for assistance to a livery keeper, and rode that twelve miles in a pouring rain, through unfathomed mud, in an open buggy with a broken-winded horse. Reaching the place we found that the memory of our subscriber had passed away, but on finding the "oldest inhabitant" he informed us that Dr. L. was employed as a tallyman in a flour warehouse on Water Street, New York. That was satisfactory, and we thought ourselves well repaid.

The second individual still remained a burden on our peace of mind. We thought we would seek him out also, but it occurred to us that a journey would be expensive. We would write to the Post-Master! Accordingly we addressed the following line to him:

"P. M., at Slogo + Roads:

"Dear Sir,

"Has Dr. —— —— 'a local habitation and a name' in your place?

"Respectfully, etc."

By return mail (oh model of promptness!) came the answer:

"Slogo + Roads.

"Dear Sir:

"Dr. —— —— *has* a '*local habitation*' here, but no '*name*' as a physician.

"Respectfully,

"—— —— P. M."

That accounts for it all thought we!

